# Consumption patterns and key motivational drivers: an analysis of food behavior in Spanish households

**DOI:** 10.3389/fnut.2025.1526957

**Published:** 2025-06-23

**Authors:** Beatriz Beltrán-de-Miguel, Susana del-Pozo-de-la-Calle, Carmen Cuadrado-Vives, Emma Ruiz-Moreno

**Affiliations:** ^1^Department of Nutrition and Food Science, Faculty of Pharmacy, Complutense University of Madrid, Madrid, Spain; ^2^Department of Chronic Diseases, National Centre for Epidemiology, Instituto de Salud Carlos III, Madrid, Spain; ^3^Consortium for Biomedical Research in Epidemiology and Public Health (CIBERESP), Madrid, Spain

**Keywords:** feeding behavior, food preferences, food consumption patterns, food consumption motivations, life style, food choice

## Abstract

**Background/objectives:**

Based on data collected in a market study, this analysis examines the food consumption patterns of Spanish households and the main individual motivations behind the use of these foods in each consumption occasion.

**Methods:**

A descriptive cross-sectional observational study was conducted using the “Household Usage Panel” (2023). Individual food consumption at home was recorded through an online survey over the course of a full week, twice a year, throughout the year. For each consumption occasion, the primary reason for the food choice was also recorded, selected from predefined options: “product was available,” “for health,” “by habit,” “taste,” “for pleasure/I felt like it,” and “quick/easy to prepare.”

**Results:**

The sample included 5,207 individuals (4,000 households), representative of the Spanish population, excluding the Canary Islands, Ceuta, and Melilla. The food groups used by more than 80% of the panelists include cereals and cereal products, vegetables, meat, fish, milk and dairy products, and fruit. For all products combined, 80.2% of consumers reported that, on at least one consumption occasion, the primary reason for use was “for taste,” 77.1% “for pleasure/I felt like it” followed by “health” (68.4%), “product was available” (59.2%), “habit” (55.3%), and “quick/easy to prepare” (47.6%). For each food group, the primary reason for use varied by occasion, with “for taste” and “for pleasure/I felt like it” chosen by a higher percentage of consumers. An exception was found with the fruit and olive oil groups, where the main reason for consumption chosen by a larger percentage of consumers on some occasions was health (57.4 and 30.8%, respectively).

**Conclusion:**

Understanding the motives behind food consumption in Spanish households helps us better grasp specific dietary behaviors. Taste and pleasure have been identified in this study as key factors in food choices at home. These insights suggest the need for a renewed approach to food policies and interventions that promote healthy eating, with the potential for lasting improvements in dietary quality over the medium to long term.

## 1 Introduction

In Spain, the food system ensures a varied and sufficient supply of food to meet the vital needs of the population (1). However, while the availability of food is undoubtedly an essential factor, it does not guarantee appropriate eating behavior or optimal nutritional quality in the diets consumed by individuals. Multiple factors—inherent to the individual (biological, psychological, conscious, and unconscious), to the foods themselves (composition, presentation, sensory attributes), or the environmental context (family, sociological, economic, environmental)—interact in complex ways to explain eating behavior (what, why, how, and when we eat). This behavior directly influences the nutritional quality of the diet and, consequently, health (2).

Among the individual factors that positively influence food choices are nutrition knowledge and the skills to manage a proper diet. Therefore, nutrition education, grounded in scientific evidence, constitutes a key component of current public health and health literacy strategies (3). However, several studies also show that nutrition knowledge is not always the primary factor determining individuals’ food choices. There are other, often more important, reasons and motivations that influence the acquisition, storage, preparation, cooking and consumption of food (4–6). It is essential to explore and delve deeper into these motivations to design more effective food literacy strategies.

In Spain, scientific studies have provided valuable information on dietary patterns and their nutritional implications in representative population samples, both nationally and in specific age subgroups (7–9). However, few publications in scientific databases analyze motivations for food use and their relationship with diet quality that can be extrapolated to the national level (10). Due to the lack of information, scientists are considering the use of data from market studies (on food purchasing and use) as a first resource for exploring the complexity of eating behavior, including aspects such as the motivations for food use (11).

Since 1990, the Spanish Ministry of Agriculture has published the “Household Food Consumption Panel,” based on data provided by Taylor Nelson Sofres S. A. U., a company specialized in market studies and public opinion surveys. The objective of this panel is to understand the direct demand for food consumption in Spanish households by systematically collecting and analyzing information on what Spaniards purchase for home consumption, how much they spend, and when and where they make their purchases. This study also investigates the differences in purchasing habits based on geographic location and other factors characterizing Spanish families. The availability of this updated data has allowed the study of food consumption trends in Spanish households over the last three decades and has indirectly estimated the implications of these changes on the nutritional quality of the diet (7, 12, 13).

In 2016, this company specialized launched a new market study, to be conducted annually, which aims to complement the information provided by the Household Food Consumption Panel by analyzing other aspects of eating behavior, including the motivations or reasons that lead individuals to choose one food from their pantry over another for their daily diet. Although the Ministry of Agriculture has released a preliminary report based on the data from this “Household Usage Panel “the detailed findings have not yet been published. We believe that a scientific and nutritional analysis of these data could have a significant impact on public health, as other authors have already suggested (14).

With this proposal, this article aims to descriptive analyze the food consumption patterns of Spanish households and the main individual motivations for using these foods in each consumption occasion, based on the data collected in the market study “Household Usage Panel.”

## 2 Materials and methods

A descriptive, cross-sectional observational study was conducted to analyze food consumption patterns and the reasons for food use based on the “Household Usage Panel” (2023), prepared by Taylor Nelson Sofres S. A. U. (Kantar Worldpanel), which meets the appropriate accreditations for companies of this type. The sample included 4,000 panelist households, representative of the Spanish population (excluding the Canary Islands, Ceuta, and Melilla). This subsample was drawn from the purchasing panel of 12,500 households, which is also used for the development of the Food Consumption Panel of the Spanish Ministry of Agriculture, which analyses household food purchases quantitatively (15).

The participating households reported, through online questionnaires, on the foods consumed at home and/or those taken from home for consumption outside. Data collection was distributed over the 52 weeks of the year. A responsible member of each household identified the food consumption carried out by each family member over a full week, twice a year, accumulating a total of 8,000 annual questionnaires, ensuring temporal representativeness across the 52 weeks.

- The time of consumption (breakfast, mid-morning, lunch, dinner, etc.).
- The method of food preparation (grilling, baking, boiling, frying, etc.).
- The specific dish or recipe (for example, omelet, pasta with tomato sauce).

Additionally, the primary reason (only one) for the use of each food by each family member during a particular consumption occasion was recorded, selected from a predefined list of options. Only one designated household member completes the questionnaire, reporting the consumption and motivations for all other household members participating. These reasons have been selected and grouped in the following way:

- Product was available
- For health
- By habit
- Taste
- For pleasure/I felt like it
- Quick/easy to prepare

Quality controls were implemented in the collected data, requiring confirmation of the products and consumers before closing the questionnaire.

The acquired data by the research team from the Kantar Worldpanel on the use of foods from the pantries of the panelists, were processed by first grouping the foods into categories. These food categories were later re-grouped by our research team according to the classification proposed by Moreiras et al. (16). The final classification consisted of the following food groups:

- Cereals and cereal products
- Vegetables
- Fruits
- Nuts
- Legumes
- Sugars
- Oils and fats
- Dairy
- Eggs
- Meat and meat products
- Fish and seafood
- Alcoholic beverages
- Non-alcoholic beverages
- Elaborated dishes
- Industrial and homemade sauces
- Spices and condiments
- Snacks

For each food and food group reported in the usage panel, the percentage of consumers in the total sample was calculated. In addition, for each item, the average number of times used per week per consumer was recorded, as well as the main reason for consumption.

These consumption occasions were recorded as seven different times of the day, including breakfast, mid-morning, lunch, afternoon snack, dinner, after dinner and takeaway.

## 3 Results

In 2023, a total of 5,207 individuals participated, corresponding to the 4,000 panelist households. Of these, 48.9% were men and 51.1% were women, with a diverse age distribution. The results indicate that 14% of the sample was under 15 years old, 10.6% were between 15 and 24 years old, and 75.7% were over 24 years old.

Table 1 shows the percentage of the total population surveyed (n = 5,091) who used food or beverages on occasion during the recorded period, as well as the average of occasions per week on which products were used. For all foods, 76.3% of the sample stated that, on some occasion, the main reason for using the food was its taste; 74.3% cited “for pleasure/I felt like it,” followed by “health” (65.2%), “product was available” (59.2%) and finally “quick or easy to prepare,” which was mentioned by almost 50% of the population. In relation to beverages, the main reason for use mentioned by a higher percentage of the sample was taste (57.5% of the reported taste as the main reason for use on some occasions), followed by “habit” (48.1%).

**Table 1:** Reasons for food and beverage consumption.

Food	Consumers (%)	Average occasions in a week per consumer	Reason for using on a particular occasion of consumption					
			Product was available (%)	For health (%)	By habit (%)	Taste (%)	For pleasure/I felt like it (%)	Quick/easy to prepare (%)
Total products	100	20.6	59.0	68.4	66.4	80.2	77.1	49.5
Total foods	99.7	18.7	59.2	65.2	55.3	76.3	74.3	47.6
Total beverages	98.6	13.6	0.0	36.8	48.1	57.5	42.8	10.2

Tables 2–6 show the percentage of the total population surveyed who used food groups or individual foods during the period recorded, as well as the average of occasions on which these products are used per week. Overall, the food groups that are used by more than 80% of the panelists are: vegetables (91.9%), meat (89.3%), fish (83.1%) and fruit (84.1%). Looking at all the foods, bread (81.6%) and milk (80.2%) stand out as the main foods chosen by more than 80% of the sample. This suggests that the consumption of the cereals and milk groups also exceeds this percentage, although the Household Usage Panel does not provide us with the total figure. The number of occasions per week on which these two foods, bread, and milk, are consumed is remarkably high compared to other products (6.2 and 6.5 respectively).

**Table 2:** Reasons for cereals and derivates, vegetables, fruits, nuts and legumes consumption.

Food	Consumers (%)	Average occasions in a week per consumer	Reason for using on a particular occasion of consumption					
			Product was available (%)	For health (%)	By habit (%)	Taste (%)	For pleasure/ I felt like it (%)	Quick/easy to prepare (%)
Cereals and cereal products								
Rice	54.7	1.5	20.0	10.6	8.2	27.1	16.7	5.9
Pastries	43.7	3.0	28.7	2.6	9.1	29.1	38.5	6.2
Breakfast cereals	26.7	3.2	16.1	22.9	15.9	33.6	20.1	9.4
Cookies	47.2	3.4	20.3	9.8	20.0	36.0	32.5	10.0
Bread	81.6	6.2	26.8	18.6	30.8	34.8	28.5	13.9
Pasta	67.0	1.7	22.8	8.1	7.2	26.8	20.0	10.1
Vegetables	91.9	6.6	35.2	42.2	21.6	49.3	37.5	16.3
Garlic	41.6	2.6	18.0	19.0	12.8	29.1	13.7	2.9
Onion	62.4	3.3	21.5	26.5	13.4	32.1	18.6	5.1
Potato	74.1	2.9	22.5	19.3	11.5	31.1	25.5	6.6
Pepper	43.5	2.2	18.3	24.3	10.0	25.3	18.4	3.7
Tomato	62.3	3.5	21.0	36.6	12.4	31.2	19.4	7.6
Carrot	44.2	2.1	18.1	31.2	9.4	23.6	13.4	3.0
Lettuce/Escarole and similar	44.0	2.4	18.4	37.4	9.7	21.8	14.8	7.2
Fruits	84.1	7.5	28.3	57.4	14.6	35.7	29.3	8.5
Kiwi	20.6	2.6	12.6	56.0	7.1	19.1	13.1	2.6
Mandarin	26.6	2.7	20.1	50.9	6.7	20.3	16.9	3.4
Apple	40.6	2.5	19.4	51.4	7.3	17.4	13.3	3.7
Orange	24.0	3.0	17.9	48.1	8.5	21.1	16.5	2.3
Banana	47.1	2.7	20.0	45.1	9.0	22.5	14.4	5.7
Nuts	17.4	2.6	13.6	27.3	7.8	18.6	32.5	3.1
Legumes	56.9	1.6	22.5	18.9	6.6	27.1	16.8	5.1
Chickpeas	26.7	1.2	20.0	15.5	5.3	24.1	14.3	4.3
Lentil	35.4	1.2	20.1	18.8	5.2	23.6	14.0	3.5
Beans	16.1	1.2	21.1	12.1	6.1	22.8	14.0	4.8

**Table 3:** Reasons for sugars, oils, and fats consumption.

Food	Consumers (%)	Average occasions in a week per consumer	Reason for using on a particular occasion of consumption					
			Product was available (%)	For health (%)	By habit (%)	Taste (%)	For pleasure/ I felt like it (%)	Quick/easy to prepare (%)
Sugars								
Cocoa cream	5.0	1.7	15.1	2.9	6.7	27.6	35.8	5.3
Chocolates	15.2	2.3	11.4	3.8	5.9	20.5	54.8	2.1
Jams/confitures	22.7	3.0	12.7	11.5	15.1	38.3	29.6	3.4
Soluble cocoa/instant	31.2	4.7	0.0	4.1	24.6	42.8	14.4	2.8
Oils and fats								
Olive oil	61.0	6.2	27.5	30.6	19.1	38.2	18.7	7.4
Sunflower oil	10.1	2.6	31.3	7.0	15.4	28.1	9.2	6.1
Margarine	15.9	3.0	15.8	10.0	19.5	33.3	20.3	5.2
Butter	15.7	2.4	14.8	6.4	16.6	32.5	25.7	4.4

**Table 4:** Reasons for dairies, eggs, meat and meat products and fish and seafoods consumption.

Food	Consumers (%)	Average occasions in a week per consumer	Reason for using on a particular occasion of consumption					
			Product was available (%)	For health (%)	By habit (%)	Taste (%)	For pleasure/ I felt like it (%)	Quick/easy to prepare (%)
Dairy								
Dairy desserts	29.3	2.6	16.0	8.5	9.1	28.3	44.3	4.9
Cheeses	64.0	2.8	20.2	12.8	10.2	36.0	27.0	11.1
Yoghurt and fermented milks	60.1	3.9	19.0	38.2	17.6	29.3	25.3	7.7
Liquid milk	80.2	6.5	0.0	23.8	39.0	39.1	14.9	4.6
Whole milk	28.0	5.4	0.0	16.6	36.6	38.7	14.2	4.4
Skimmed milk	25.1	5.5	0.0	29.7	32.2	33.7	14.5	4.2
Semi-skimmed milk	40.0	5.5	0.0	22.2	38.5	36.7	11.5	3.5
Eggs								
Eggs	79.9	2.6	23.6	15.5	10.6	30.5	25.9	11.5
Meat and meat product								
Meats	89.3	3.9	30.9	19.5	10.6	41.2	35.6	13.8
Beef	38.8	1.6	19.1	10.0	4.9	28.1	23.2	5.5
Pork	61.6	2.0	25.0	7.5	6.9	31.9	24.8	9.0
Lamb meat/Goat meat	6.5	1.2	16.9	5.7	1.7	24.7	23.4	1.2
Poultry	72.1	2.1	22.4	18.4	6.9	31.1	23.8	7.9
Meat products	75.4	3.3	26.0	13.1	11.4	40.8	29.0	13.1
Charcuterie	65.2	2.6	23.5	8.9	9.1	38.6	26.7	8.8
Cold meats and stews	42.5	2.1	20.0	13.8	8.3	30.7	19.9	13.5
Sausages	21.5	1.2	21.5	2.6	2.9	23.4	21.0	12.3
Fish and seafood	83.1	3.0	25.5	26.6	7.3	37.3	30.0	10.2
Fresh and frozen fish	64.1	1.9	20.4	27.9	4.0	26.9	21.7	5.9
Fresh and frozen seafood	23.8	1.4	13.7	8.3	4.2	24.9	26.9	2.5
Fish and seafood canned	47.8	1.8	19.1	15.1	6.1	30.9	19.8	8.6

**Table 5:** Reasons for alcoholic beverages, non-alcoholic beverages, and vegetable drinks consumption.

Food	Consumers (%)	Average occasions in a week per consumer	Reason for using on a particular occasion of consumption					
			Product was available (%)	For health (%)	By habit (%)	Taste (%)	For pleasure/I felt like it (%)	Quick/easy to prepare (%)
Alcoholic beverages								
Distilled alcoholic beverage	2.2	2.0	0.0	0.4	3.4	27.1	48.2	0.0
Beer with alcohol	16.7	2.7	0.0	2.1	9.1	23.5	46.8	0.9
Wines	21.7	3.6	0.0	4.1	15.7	25.8	42.1	2.0
Non-alcoholic beverages								
Bottled water	36.6	8.8	0.0	31.6	30.6	21.2	9.6	3.2
Coffees	62.8	6.9	0.0	5.4	31.3	38.2	20.4	4.0
Infusions	18.7	4.7	0.0	23.9	18.1	26.7	26.6	3.9
Carbonated soft drinks	27.3	4.0	0.0	2.6	12.3	29.8	31.6	1.8
Homemade juices	14.5	3.5	0.0	47.8	12.5	18.2	22.9	1.2
Vegetable drinks								
Milk substitute 100% vegetal	2.4	2.2	7.5	40.1	14.9	27.7	17.1	5.9
Vegetable drinks	7.4	5.3	0.0	39.7	20.5	33.5	14.5	5.0

**Table 6:** Reasons for elaborated products, industrial and homemade sauces, spices and condiments and snacks consumption.

Food	Consumers (%)	Average occasions in a week per consumer	Reason for using on a particular occasion of consumption					
			Product was available (%)	For health (%)	By habit (%)	Taste (%)	For pleasure/I felt like it (%)	Quick/easy to prepare (%)
Elaborated dishes							
Pre-prepared dishes	55.8	2.0	15.8	3.8	3.9	19.0	17.3	16.3
Dehydrated dishes	2.3	1.2	18.9	2.3	1.3	22.0	11.5	13.4
Room temperature dishes	12.0	1.3	9.9	4.7	2.0	13.5	11.2	13.0
Refrigerated dishes	35.1	1.6	14.7	1.8	3.2	16.3	14.1	14.8
Frozen dishes	22.6	1.4	10.6	2.5	3.7	13.9	12.4	9.0
Dishes not specified technique	10.1	1.3	13.4	4.6	2.4	19.2	17.4	13.1
Industrial and homemade sauces	63.7	2.3	18.6	6.8	9.0	36.8	20.4	8.9
Industrial sauces	52.2	2.0	18.7	5.1	8.4	36.2	18.4	9.4
Industrial fried tomato	33.6	1.6	17.3	4.5	8.3	32.1	13.6	10.0
Industrial ketchup	9.6	1.3	12.7	1.7	5.8	31.3	15.9	3.9
Industrial mayonnaise	18.7	1.5	17.2	2.5	6.2	30.0	15.6	5.0
Industrial mustard	4.5	1.3	11.9	2.3	5.1	25.3	15.6	3.6
Homemade sauces	26.9	1.6	11.6	7.8	6.0	26.6	17.9	3.7
Homemade fried tomato	13.5	1.4	11.1	7.6	6.1	25.8	15.1	3.4
Homemade mayonnaise	4.4	1.3	10.9	7.3	6.5	25.2	20.4	3.0
Spices and condiments							
Bouillon cubes	17.9	2.2	20.3	5.6	13.0	35.5	10.2	4.6
Spices and herbs	26.7	2.5	4.2	5.6	5.3	11.9	5.7	0.6
Vinegars	17.5	2.5	5.1	8.1	5.3	11.8	6.0	1.0
Snacks								
Olives and pickles	26.1	2.0	20.2	10.8	6.3	30.1	27.8	4.8
Chips and snacks	19.6	1.7	17.6	2.2	4.8	26.2	40.9	5.1
Chips	16.9	1.6	17.5	1.6	4.2	25.7	38.4	5.3
Other snacks	6.3	1.4	11.8	2.5	3.4	23.7	42.2	2.6

### 3.1 Cereal and cereal products

Of the cereals and cereal products foods (Table 2), we can highlight the use of bread, because 81.6% of the population includes this food in their household diet, a value considerably higher than the number of consumers of pasta (67%), rice (54.7%), cookies (47.2%) and pastries (43.7%). Breakfast cereals were included in the household diet of 26.7% of the total population. As mentioned above, the number of occasions bread was used per week (6.2) was among the highest. Other foods in the group such as cookies and breakfast cereals were consumed on 3.2 and 3.4 occasions per week, respectively. In the foods included in the cereals and cereal products group-shown in Table 2, “taste” was the reason for use chosen by a higher percentage of the sample on at least one occasion, except for pastries where “for pleasure/I felt like it” occupied the first place (38.5%). The “availability” of the product at home was sometimes considered as the main reason by more than 20%, particularly for pastries (28.7%). Use “for health” reasons only exceed this percentage (22.9%) of the sample for breakfast cereals. “Taste” was often chosen as the main reason, especially for cookies (36.0%) and bread (34.8%). “By habit” as the primary reason was notably high for bread, reaching 30.8%. The reason “quick or easy to prepare” was the least mentioned for this group of foods.

### 3.2 Vegetables

Among the food groups that the survey covered globally, it is worth highlighting that vegetables were used by the highest percentage of panelists, according to the market survey data (91.9%). There were 6.6 average occasions in a week per consumer. Potatoes, along with onions and tomatoes, were the most selected (74.1, 62.4, and 62.3% respectively) to be included in their diet. Other foods selected by more than 40% of consumers include carrots (44.2%), lettuce (44.0%), peppers (43.5%) and garlic (41.6%). The foods in this group that were selected most often per week were tomato (3.5) and onion (3.3).

The main reason for using vegetables, according to the highest percentage of consumers, was taste (49.3%) followed by “health” (42.2%), “for pleasure/I felt it like” (37.5%) and “product was available” (35.2%). Among the specific foods, garlic (29.1%), onion (32.1%), potato (31.1%) and tomato (31.2%) were chosen by the highest percentage of the sample for “taste,” while lettuce/escarole and similar (37.4%), tomato (36.6%), carrot (31.2%), were selected “for health” reasons (Table 2).

### 3.3 Fruits

Fruit was another of the food groups mentioned overall in the survey, which stood out for being chosen by a very large percentage of participants (84.1%). There were 7.5 average occasions in a week per consumer. Table 2 includes data on those fruits consumed by more than 20% of the sample: banana (47.1%), apple (40.6%), mandarin (26.6%), orange (24.0%) and kiwi (20.6%). Among the selected foods in this group, the number of occasions used per week was very similar between them, ranging from 2.5 (apple) to 3 (orange). The main reason for use of this group, to which the largest number of consumers referred was “health” (57.4%) followed by “taste” (35.7%). This trend was repeated for each of the fruits included.

### 3.4 Nuts

The number of occasions on which nuts were chosen by 17.4% of respondents was 2.6 per week. 32.5% of nut consumers chose them for “pleasure/I felt like it” as their main reason for consumption on some occasion, followed by “for health” (27.3%), and “taste” (18.6%) (Table 2).

### 3.5 Legumes

The legumes group was consumed by 56.9% of the sample, with lentils being the most included in the diet (35.4%), followed by chickpeas (26.7%) and beans (16.1%). The number of occasions on which pulses were used per week was 1.6 and was the same for the three types of legumes studied (1.2).

“Taste” (27.1%) was the main reason selected by the largest number of participants for choosing this food group on some occasion of consumption, especially in chickpeas, where 24.1% of consumers mentioned it as a reason, followed by “product was available” (22.5%), which was mainly mentioned in beans (21.1%). The next reason was “for health,” cited by 18.9% of the consumers of pulses (Table 2).

### 3.6 Sugars

For the sugars group, soluble/instant cocoa was chosen by the most participants (31.2%) and on the highest number of occasions per week (4.7). About the other foods, jams or other foods, jams or confitures were chosen by 22.7% of the sample, with an average of 3 occasions per week. Chocolates were chosen by 15.2% of participants (with an average of 2.3 occasions) and cocoa cream by 5% (1.7 occasions). The main reasons for use mentioned by the highest number of participants on some occasion in this group were “for pleasure/I felt like it” in the case of chocolate (54.8%) and cocoa cream (35.8%), and “taste” in soluble cocoa/instant (42.8%) and jams/confitures (38.3%) (Table 3).

### 3.7 Oils and fats

Within this group, olive oil was chosen by the highest number of participants (61%) and was use on an average of 6.2 occasions per week. Margarine, butter, and sunflower oil were used by 15.9, 15.7 and 10.1% of the participants, respectively, with the number of occasions per week being similar between them, ranging from 2.4 (butter) to 3 (margarine). The main reason for using olive oil, to which the highest percentage of consumers referred, was “taste” (38.2%) and the same was true for margarine (33.3%) and butter (32.5%). For the choice of sunflower oil, the main reason given was “product was available” (31.3%), with “taste” in second place (28.1%). For olive oil, “for health” was the reason given by 30.6% of consumers, and for margarine and butter, “pleasure/I felt like it” (20.3 and 25.7%, respectively) was the second reason given after taste (Table 3).

### 3.8 Dairy

Of the foods in the dairy group, milk varieties (80.2%) (whole milk (28%), skimmed milk (25.1%) and semi-skimmed milk (40%)), cheeses (64%), yoghurts and fermented milks (60.1%) and dairy desserts such as flans, creams, custards, etc., were selected for this analysis, as they were consumed by a greatest number of participants (29.3%) (Table 4). As mentioned at the beginning of this section of the results, the number of occasions on which milk was used per week (6.5) was greater than for other foods in this group. The main reasons for use referred by the highest percentage of consumers of milk and its different varieties—whole, skimmed, and semi-skimmed—were “taste” (39.1, 38.7, 33.7 and 36.7%) and “by habit” (39, 36.6, 32.2 and 38.5%). In the case of yoghurts and fermented milks, the main reason mentioned by more participants was “for health” (38.2%); in the case of dairy desserts, it was “for pleasure/I felt like it” (44.3%) and in the case of cheeses, it was “taste” (36%).

### 3.9 Eggs

Eggs were used by almost 80% of the participants on an average of 2.6 occasions per week, with “taste” being the main reason for consumption selected on some occasion by a highest percentage of the sample (30.5%), followed by “for pleasure/I felt like it” (25.9%) and “product was available” (23.6%) (Table 4).

### 3.10 Meat and meat products

The subgroup of meats was chosen by a high percentage of consumers (89.3%), with an average of 3.9 occasions per week. The subgroup of meat products was selected by 75.4% of consumers, with a similar average of 3.3 occasions of consumption per week, and charcuterie stood out with 65.2% of consumers. The type of meat chosen by the highest percentage of participants was poultry (72.1%) and pork (61.6%), with a similar average number of occasions per week (2.1 and 2, respectively).

The main reason for using meat referred to by the highest percentage of consumers on some occasion was “taste” (41.2%), which was the primary reason for all types of meat (beef (28.1%), pork (31.9%), lamb/goat (24.7%), and poultry (31.1%)). This was followed by “for pleasure/I felt like it” (35.6%) and “because it is available” (30.9%). The main reason for use meat products selected for a highest percentage of consumers also was “taste,” both for charcuterie (38.6%) and cold meats and stews (30.7%) and sausages (23.4%) (Table 4).

### 3.11 Fish and seafood

Just like group of meats, fish and seafood was a group of foods chosen by a high number of consumers (83.1%) with an average of 3 occasions per week. Within this group, fish was selected by 64.1% of the participants, with an average number of occasions per week of 1.9, while seafood was chosen by only 23.8% of participants, with an average of 1.4 occasions per week. Canned fish and seafood were chosen by 47.8% of the participants, with an average of 1.8 occasions per week.

In general, the main reason for using this group for consumers was “taste” (37.3% of consumers selected this reason on some occasion), followed by “for pleasure/I felt like it” (30%) and “for health” (26.6%). With specific reference to fish, it is important to note that the main reason given by the highest percentage of participants on some occasion was “health” (27.9%), while for seafood the main reason was “for pleasure/I felt like it” (26.9%), and for canned food it was “taste” (30.9%) (Table 4).

### 3.12 Alcoholic beverages

Household consumption of alcoholic beverages was focused on beer—chosen by 16.7% of the participants—and wine—chosen by 21.7% of the participants. Distilled alcoholic beverages were selected by 2.2% of the participants. The average number of occasions per week per consumer were 3.6 for wine, 2.7 for beer, and 2 for distilled beverages. The main reason for use, selected for a highest percentage of consumers, for both wine as well as distilled alcoholic beverages, was “for pleasure/I felt like it,” with between 42.1% (for wine) and 48.2% (for distilled beverages) (Table 5). Please note that these are general population averages and the variability between age groups has not been considered for this publication.

### 3.13 Non-alcoholic beverages

Among the non-alcoholic beverages included in this study, coffee was the one selected by the highest percentage of participants (62.8%) and with a high frequency of occasions, averaging 6.9 per week. Bottled water was selected by 36.6% of the participants, averaging 8.8 occasions per week, and carbonated soft drinks were chosen by 27.3% of the participants on 4 occasions per week.

Homemade juices were selected by 14.5% of the participants, averaging 3.5 occasions of use per week, and their main reason for use, declared by a highest percentage of consumers, was “for health” (47.8%). This was also the main reason for using bottled water (31.6%). On the other hand, “taste” was the main reason for using coffee (38.2% of consumers selected this motive as principal on some occasion), followed by “habit,” which was cited by 31.3% (Table 5).

Regarding vegetable drinks, 7.4% of participants used them, on average 5.3 times per week. “For health” was cited by 39.7% of consumers as the main reason for using them on some occasion, while “taste” was cited by 33.5% (Table 5).

### 3.14 Elaborated dishes

The group of pre-prepared dishes was consumed by 55.8% of the participants, with an average of 2 occasions per week. The main reason for use of these foods, selected by the highest percentage of consumers, on some occasion, was “taste” (19%), followed by “for pleasure/I felt like it” (17.3%), “quick/easy to prepare” (16.3%), and finally “product was available” (15.8%).

Within this group, the most selected items for use were the refrigerated dishes (35.1% of the participants), averaging 1.6 occasions per week; frozen dishes were chosen by 22.6%, with an average of 1.4 occasions per week, and room temperature dishes by 12%, averaging 1.3 occasions per week. The main reason for use selected by the highest percentage of consumers on some occasion in each of these subgroups followed the same trend as this for the overall pre-prepared dishes (Table 6).

### 3.15 Industrial and homemade sauces

A total of 63.7% of the participants used sauces, industrial and homemade, on an average of 2.3 occasions per week. Among the sauces included in this study, industrial sauces were used by 52.2% of the participants, averaging 2 occasions per week, while homemade sauces were chosen by 26.9% of the participants, averaging 1.6 consumption occasions per week. Fried tomato sauce, industrial and homemade, was the sauce selected by the highest percentage of participants (33.6 and 13.5%, respectively), consumed between 1.6 and 1.4 occasions per week. Mayonnaise followed with 18.7 and 4.4% of consumers (industrial and homemade, respectively).

Among the main reasons for use of industrial and homemade sauces remark “taste” selected by 36.8% of consumers on some consumption occasion, followed by “for pleasure/I felt like it” (20.4%), and “product was available” (18.6%) (Table 6).

### 3.16 Spices and condiments

Included in this group, spices and herbs were reported to be used by 26.7% of the sample (Table 6). Among the main reason for use, 11.9% of spice consumers stated that they used them on some occasion for “taste.”

### 3.17 Snacks

Regarding snacks, the percentage of consumers of olives and pickles were notable (26.1% participants), with an average of 2 occasions per week, slightly lower for chips and other snacks at 19.1% of consumers and 1.7 occasions per week. The main reason for use focused on “taste” (30.1% of consumers of olives and pickles and 26.2% of consumers of chips and other snacks selected this use motive on some occasion), “for pleasure/I felt like it” (27.8 and 40.9%, respectively), and “product was available” (20.2 and 17.6%, respectively) (Table 6).

## 4 Discussion

The data used in this study originate from a market survey conducted on a representative sample of the Spanish population (excluding the Canary Islands, Ceuta, and Melilla), offering a valuable resource to expand the limited information available on food usage motivations in Spanish households, starting from their pantry supplies. Furthermore, this type of analysis, combined with the assessment of other aspects of food behavior, presents an opportunity to refine food literacy strategies for the population, which must account for existing social determinants, which are, at times, challenging to change. The annual and recurring nature of this survey also provides a robust foundation to investigate trends and changes in food habits among Spanish households over time.

The results obtained in this study indicate that the percentage of consumers of the different food groups conforms basically to the traditional Mediterranean dietary pattern in Spain (16). This reflects a varied diet, in which food groups such as fruits, vegetables, meats, fish, and eggs, together with specific products such as bread and milk, are chosen by a large percentage of the population, with the highest frequency of consumption recorded in the survey for the groups of fruits and vegetables and for bread and milk.

Olive oil remains the preferred culinary fat in Spanish households, significantly surpassing sunflower oil, despite the notable price increase in recent years (17, 18). It is important to note that the real use of the product could be underestimated. The experience of the research team, aligned with that of other authors (19), shows that individuals when questioned about what they eat daily often forget or omit information about culinary fats used in food preparation. Within the Mediterranean pattern, wine also plays a significant role, being the most consumed alcoholic beverage in households to accompany meals. Similarly, this product could be underestimated by considering the average values for total population.

It is noteworthy that, although legumes are a fundamental pillar of the Mediterranean dietary pattern, just over half of the participants in this study reported consuming them, with a weekly consumption frequency of less than two occasions. These results align with other studies such as ANIBES in the Spanish population (20) and the data from the Food Consumption Panel by MAPA (12). The vegetables group includes a wide range of foods, as mentioned earlier in the results section. Notable items in this group include potatoes, onions, and tomatoes, with the latter two serving as essential ingredients in sofrito, an iconic recipe in Mediterranean cuisine (21). Fruits reflect their daily presence in the diet. The most consumed fruits were those available year-round, while other fruits were selected by a smaller percentage of participants. This rotation in the types of fruit consumed throughout the week positively contributes to dietary variety, promoting a more balanced and diverse intake (22). Only a small percentage of participants included nuts in their diet, which may indicate that, despite their inclusion in a traditional Mediterranean dietary pattern, this food group is not widely consumed in our country. However, the average weekly consumption among consumers is notable, suggesting that this group is becoming more established in the diets of those who do consume it.

While the Mediterranean dietary pattern promotes the consumption of fresh or minimally processed foods, the pre-prepared dishes group was selected by more than half of participants, with an average of two occasions per week. This figure clearly reflects the global trend toward increased consumption of ready-to-eat or quick-preparation foods (23) and raises questions about its impact on the population’s nutritional status, warranting further in-depth study (24).

Based on the results of this study, it can be generally stated that the primary reason for consuming a food varies considerably; consumers may select a food for a different primary reason on each occasion. This could explain why the percentages of consumers choosing a food for various reasons, such as “taste,” “by habit,” or “for pleasure,” were similar. An example is bread, which was chosen on some occasions by 34.8% of consumers for its taste, 30.8% out of habit, and 28.5% for pleasure. This finding highlights the complexity of food behavior (25), which can vary both among different individuals and within the same individual, depending on the consumption occasion. Additionally, it is worth noting that the study does not capture the full range of possible motives, as the question about the primary reason for consumption is closed-ended, preventing participants from mentioning other reasons that might influence their choices.

Among the main reasons for food use overall, “for taste” and “for pleasure/I felt like it” stand out. Although presented as distinct motives in this survey, both concepts are related to the experience of enjoyment in eating. ‘Taste,” which can be broadly understood as “flavour,” specifically refers to the sensory perception experienced when a food is eaten. On the other hand, “pleasure” has a broader meaning related to food enjoyment, encompassing three interrelated dimensions. The *sensory dimension* refers to the immediate sensations produced by the food, with taste being an essential component; the *interpersonal dimension* encompasses the social and cultural context in which food is shared, such as interactions with family or friends during meals; and lastly, the *psychosocial dimension* includes the cognitive and emotional associations that people link to food, such as memories and cultural meanings attached to certain foods (26).

The primary value of “taste,” linked to the sensory perception of food, is evident across nearly all food groups, including cereals, legumes, dairy, meats, fish, eggs, sauces, and even vegetables. However, within the fruit category, health emerges as the main reason for selection among the largest percentage of consumers. This awareness on the fruits health benefits appears to be deeply ingrained in the sample, surpassing even “taste” in the percentage of consumers declared on some occasion“for health” as the main motive for use them. Similarly, olive oil was chosen by 30.6% of consumers ‘for health’ and vegetable drinks by 39.7%. The promotion of fruit and olive oil consumption by international organizations has clearly influenced purchasing and consumption decisions based on health criteria (27, 28). While fruit consumption reflects a universal message about its benefits, olive oil—as a fat, a food type often carrying negative connotations—seems to have greater national momentum, likely due to its cultural significance in Spain as a major producer. However, these results also suggest that other food groups, such as legumes, have not achieved similar recognition despite national and international efforts to boost their consumption (e.g., the FAO’s International Year of Pulses in 2016) (29). In this sense, we consider that emphasizing the enjoyment of food as an “incentive message” in education actions could favor greater dietary inclusion of this food group. For example, reformulating traditional recipes or creating new preparations that make pulses more attractive could diversify their forms of consumption and increase their presence in daily meals.

“For pleasure/I felt like it” held a predominant place in the consumption of nuts, even surpassing “taste” as the main motivation selected by a higher percentage of consumers on some consumption occasion. This pattern also appeared with other foods such as chocolate, pastries, dairy desserts (custards, flans), and salty snacks, all characterized by their high content of sugar, fat, and/or salt. Acceptance of these foods is closely linked to the sensory perception of these components (30, 31) but may also relate to the satisfaction derived from consumption, as viewed from the other dimensions of pleasure, interpersonal and psychosocial.

These observations suggest that health promotion strategies should emphasize food enjoyment (31, 32). Clearly, if a food is not liked, it will not be consumed; pleasure is a crucial factor in food choice. This is especially true when considering countries where most of the population has access to enough food to meet basic needs. Increasing pleasure aspect of foods with a healthy profile is a clear target for educational intervention strategies. And, in this sense, culinary literacy could improve the palatability of the diet without compromising nutritional quality (33, 34).

Based on the results, we propose that effective food literacy interventions should focus on highlighting the sensory attributes of food, particularly taste and pleasure, as key drivers of food choices. Educational programs could emphasize the enjoyment of healthy foods by promoting their taste and sensory appeal through initiatives such as cooking classes or specialized workshops. Incorporating social and cultural aspects of food would ensure these strategies resonate with diverse audiences. Tailoring interventions to different socioeconomic groups and collaborating with local producers, chefs, and nutritionists could further help create more accessible and sustainable solutions for promoting healthy eating habits.

In this context, few food-based dietary guidelines include recommendations related to the message of “enjoying” food or other aspects of food pleasure, such as eating in company (associated to the interpersonal dimension of pleasure) and developing culinary skills (34, 35). For example, the Mediterranean Diet Foundation highlights the importance of commensality (sharing meals with others) and acquiring basic culinary skills, placing these at the base of the food pyramid (35).

When other main motives for consumption, such as “product was available, “by habit,” and “quick/easy to prepare,” are analyzed in this study, no food stands out as extensively chosen for these reasons compared to those previously discussed. These factors undoubtedly shape eating behavior, consistent with other studies (6, 10, 36) and some study consumers (but in a lower percentage than those corresponding with other motives) chose them as primary motives on some occasions. Availability of food at home influences consumption and is closely linked to grocery choices. Therefore, from a food literacy perspective, educating consumers about effective grocery planning could enhance the nutritional quality of foods available at home (37).

On the other hand, more than 30% of consumers reported that they often choose foods “by habit,” particularly in the case of bread, milk (in all varieties), and bottled water. While “quick and easy to prepare” was a main reason for some consumers on certain occasions, it did not surpass other motivations, even for pre-prepared dishes.

As noted in the review by Bandy et al., there is a significant amount of gray literature discussing the motives behind food purchases; however, there are few scientific studies rely on market surveys (10, 11). To our knowledge, no previous scientific publications in Spain have analyzed usage motives using a representative sample based on market surveys, although there are studies that examine food consumption (12, 13). This lack of previous research makes it difficult to compare our study results with existing literature.

### 4.1 Article limitations and strengths

The primary limitations of this article stem from the inherent characteristics of the market survey used as the information source. The use of this type of survey in scientific studies—originally designed for commercial purposes—brings specific limitations, as noted by other authors (11). Among these, the need for researchers to purchase data, which is often very expensive (thus limiting accessibility), the lack of control researchers has over the recruitment process, and the incomplete information provided regarding recruitment and methodology used for data processing are notable. Another limitation of this type of study, as described in the methodology, is that it relies on closed-ended responses with preselected motivations. Although these motivations may reflect the general motivations of the population, as they were selected based on previous studies conducted by the company, this is mostly true for most of the foods. Furthermore, in this case, the data provided in this paper, are population averages, which have the limitation of not taking into account differences based on factors such as age, gender, socio-economic and other factors. This is especially relevant for foods like alcoholic beverages, for example, where such differences could significantly influence consumption patterns. We intend to include more detailed analyses in subsequent publications once more data on relevant variables become available.

Nonetheless, the academic exploitation of consumer data derived from market surveys has certain strengths, which justify its increased use in academic research on health behavior in recent years. Despite the limitations mentioned above, the information obtained through this channel, when properly contextualized, can complement data collected in nutritional surveys (12). Market surveys provide data on representative samples of the population, offering more representative insights into dietary habits and allowing detailed demographic breakdowns by accessing hundreds of thousands of households. Moreover, they include information not only on food consumption but also on other variables associated with the complex realm of food behavior at every level of food management, including purchasing, food preparation, and household waste management. These data can be leveraged by researchers in nutritional epidemiology to measure dietary patterns (7), estimate nutrient intake (12) track changes in the nutritional composition of food products over time (38), model disease outcomes (39) and assess policies. The continuous periodic repetition (annual frequency) of market surveys using similar methodologies allows for the easy measurement of consumption trends over time.

## 5 Conclusion

Understanding and analyzing the motives behind food consumption within Spanish households enhances our ability to grasp the reasons behind specific dietary behaviors—What do we eat, and why do we eat it? This understanding could facilitate the design of more effective food literacy strategies that not only align with health recommendations but also consider the motivations and contextual factors influencing individual food choices. Such an approach can improve adherence to these recommendations.

In this regard, the results of this study place taste and pleasure in a privileged position as primary determinants of food choices at home. Consistent with existing literature, these findings suggest that educational strategies promoting healthy eating should emphasize the palatable aspects of foods, rather than focusing solely on their health benefits.

In both home cooking and food service contexts, evidence indicates that taste is a dominant factor in food decision-making. Taste often takes precedence over other considerations, making it a powerful tool in promoting the consumption of nutritionally sound foods or recipes by highlighting their enjoyable attributes (the three dimensions of pleasure). Any culinary initiative aimed at improving the palatability of healthy foods can help foster and reinforce positive eating behaviors.

In summary, these observations suggest a new approach to the development of food policies and interventions aimed at promoting healthy diets, with the potential for significant and sustained improvements in dietary adherence in the medium to long term.

## Data Availability

The datasets presented in this article are not readily available because the data are the property of the funding institution. Requests to access the datasets should be directed to ER-M, e.ruiz@isciii.es.
